# Rethinking the role of biomarkers for operable non-small cell lung carcinoma: an effective collaboration with artificial intelligence algorithms

**DOI:** 10.1038/s41379-022-01167-8

**Published:** 2022-10-07

**Authors:** Esther Conde, Susana Hernandez, Fernando Lopez-Rios

**Affiliations:** 1 Pathology Department, 12 de Octubre University Hospital, Universidad Complutense de Madrid, Research Institute 12 de Octubre University Hospital (i+12), CIBERONC, Madrid, Spain; 2 Pathology Department, 12 de Octubre University Hospital, Research Institute 12 de Octubre University Hospital (i+12), Madrid, Spain

The treatment landscape of advanced non-small cell lung cancer (NSCLC) has changed tremendously in the past 20 years, with the arrival of targeted therapies and immune checkpoint inhibitors (ICIs)^1^. Despite this success in the advance setting, the 5-year survival of operable NSCLC patients is only 80% for stage I and 13–60% for stages II and III^1^, so interest has reawakened in the synergy of chemotherapy, targeted therapies and ICIs in the adjuvant and neoadjuvant settings^2,3^. The latest version of the NCCN guidelines include nivolumab and platinum-doublet chemotherapy as neoadjuvant systemic therapy, and osimertinib and atezolizumab as adjuvant systemic therapies after adjuvant chemotherapy^4^. Therefore, the current strategy is to increase the number of perioperative therapies under investigation, with large knowledge gaps concerning the best way to select patients.

The potential need to search for predictive biomarkers in all of our patients with early-stage NSCLC is a mixed blessing. On the one hand, we could really improve the overall survival of our patients, but on the other hand one might suffer decision paralysis: we will have more Therapies, more Testing (serial tissue and plasma samples, both before and after surgery) and more Technologies to schedule (the “3 Ts” conundrum). For example, as every pathologist knows, the interpretation of any immunohistochemistry (IHC) stain on large tissue sections or in consecutive specimens from the same patient is always more challenging than scoring small tumor areas or signing-out and isolated IHC report, respectively.

Few topics in clinical cancer research have been as contentious as the field of biomarkers for ICIs in patients with advanced tumors. In patients with advanced NSCLCs, the most established are probably PD-L1 expression, studied with IHC, and the tumor mutational burden, as a surrogate for neoantigen generation^5^. Notwithstanding the clinical success of ICIs, there is clearly a need for deeper multiomics biomarker efforts to incorporate, not only recent genomic perspectives (microsatellite instability and other tumor molecular variables), but also the tissue-based classification of the tumor microenviroment [i.e., the analysis of the tumor infiltrating lymphocytes (TILs) and beyond^6^]. Fortunately, the increasing use of artificial intelligence (AI) algorithms for the evaluation of immune biomarkers on tissue sections is a wonderful opportunity to address the potential biases of heterogeneity and interobserver/intraobserver variability^7^.

In this issue of *Modern Pathology*, Zens et al.^8^ provide further insights into the role of predictive immune biomarkers in operable NSCLC. Specifically, they investigated the effect of neoadjuvant chemotherapy on PD-L1 expression and CD8+ TILs density in a retrospective cohort of NSCLC patients. Although the authors acknowledge that some of their conclusions are limited precisely by the real-life nature of their design (i.e., insufficient subgroup sample size, heterogeneity of chemotherapy regimens, etc…), this well-conducted study addresses several controversial topics and provides the framework for future research.

In spite of the fact that the quest for tissue biomarkers of response to chemotherapy is now long-abandoned, in this new paradigm we need to understand the interplay between chemotherapy and ICIs. Is there increased PD-L1 expression after chemotherapy?. Considering their results (manual scoring of clone SP263 on paired samples) and their review of the literature, the authors conclude that it seems unlikely that neoadjuvant chemotherapy induces PD-L1 expression. In view of the potential importance of this conclusion, it must be emphasized that a broadly held consensus has yet to emerge (summarized in Table 1 by Zens et al.^8^).

The continuous appearance of contradictory results remains troubling, and we firmly believe that some of these inconsistencies could be resolved with the use of PD-L1 AI algorithms^9^. The finding that only 55% of the available pre-treatment NSCLC specimens had sufficient tumor content for PD-L1 testing should increase pathologists awareness on the importance of limiting diagnostic IHC, regardless of stage^10^.

Perhaps even more difficult than understanding the dynamics of PD-L1 is initially judging the predictive role of TILs in early-stage NSCLC. Is there increased CD8+ TILs density after chemotherapy?. Zens et al.^8^ evaluated CD8+ TILs per mm^2^ using a semi-automated approach with an open-source AI software. Regions of interest were manually annotated and only peritumoral stromal TILs were analyzed. In agreement with other studies, CD8+ TILs density correlated with PD-L1 expression and was significantly lower before neoadjuvant therapy when paired samples were compared. However, due to the limitations mentioned earlier, the authors did not conclude that chemotherapy increases CD8+ TILs densities. Despite these shortcomings, we await with great interest the subsequent publication of their standard operating procedure (technical manuscript in preparation) so their hypothesis could be investigated in other cohorts (see below). Although CD8+ TILs density has been identified as the most predictive variable of the response to ICIs across cancer types and multimodality biomarker strategies have been shown to improve prediction^11,12^, the use of this information in real clinical practice has remained elusive. In our opinion, the main reasons are the variety of assessment protocols [hematoxilyn and eosin (H&E) stained sections versus CD8 IHC or multiplex IHC/immunofluorescence, manual versus computer-assisted estimation, etc…]^5,13^ and the limited enthusiasm for retesting correlations or methodologies established by others. Despite the lack of harmonization, AI solutions are paradoxically re-focusing digital quantification of TILs as a predictive biomarker for ICIs in patients with advanced NSCLC, with a special interest on the spatial distribution of lymphocytes and their relationship with tumor cells^14–18^. Therefore, the data presented by Zens et al.^8^ should help implement reproducible annotation protocols for AI in operable NSCLC. This is not a trivial task, as the number of questions pathologists are asking is increasing: how many fields of view do I need to score?, shall I consider only peritumoral stromal TILs?, why not including intra-epithelial TILs?, how far from the tumor margin is too far?, why are acinar patterns more time-consuming? are squamous cell carcinomas easier to score? etc…^19^. This line of reasoning also suggests that reflex testing of immune biomarkers in early-stage NSCLC should obtain supporting data for the long-awaited TNM immune cell score^20^.

Next, the authors assessed the number and activity (presence of germinal centers) of tertiary lymphoid structures (TLS) in the resection specimens. As expected, higher number of TLS correlated with higher CD8+ TILs density. Of note, in 44 cases a different block than the one used for PD-L1 and CD8 IHC had to be selected to include adjacent normal lung, highlighting the importance of adequate sampling after neoadjuvant therapy^21^. In fact, the presence of TLS has been significantly associated with the number of fields of view analyzed^17^, a plausible explanation for its lower predictive performance than TILs^16,17^. In patients with advanced NSCLC, subsets of specific CD8+ TILs correlated with response to ICI, and they were predominantly localized in TLS^16^. Therefore, further studies in resected specimens should continue exploring the predictive role of TLS as their identification on H&E stained sections is straightforward.

**Fig. 1:**
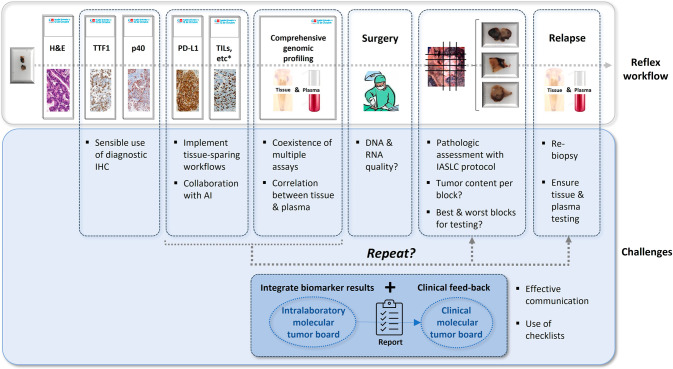
Reflex biomarker workflow for patients with operable NSCLC, candidates to neoadyuvant and adyuvant therapies. *Tissue-based predictive biomarkers for targeted therapies, ICIs or antibody-drug conjugates might require the use of several tissue sections, so the implementation of tissue-sparing protocols cannot be overemphasized. AI artificial intelligence, H&E hematoxylin and eosin, IASLC International Association for the Study of Lung Cancer, ICIs immune checkpoint inhibitors, IHC immunohistochemistry, NSCLC non-small cell lung carcinoma, PD-L1 programmed death cell ligand 1, TILs tumor infiltrating lymphocytes.

In summary, the data presented in this issue of *Modern Pathology* has the potential to refine the tools we are going to need for the next-generation of lung cancer biomarkers. We foresee that the challenges of early-stage NSCLC testing will require reflex laboratory workflows, some biomarkers interpreted with the collaboration of AI algorithms and, most importantly, healthy teamwork with effective communication (Fig. 1).
